# Adapting a dyadic exercise program to be culturally relevant for Hispanic men with prostate cancer using community engagement studio: a brief report

**DOI:** 10.3389/fpsyg.2024.1294546

**Published:** 2024-04-23

**Authors:** Meghan B. Skiba, Terry A. Badger, David O. Garcia, Floyd H. Chilton, Kerri M. Winters-Stone

**Affiliations:** ^1^College of Nursing, Advanced Nursing Practice and Science Division, University of Arizona, Tucson, AZ, United States; ^2^Mel and Enid Zuckerman College of Public Health, Department of Health Promotion Sciences, University of Arizona, Tucson, AZ, United States; ^3^College of Agriculture and Life Sciences, Department of Nutritional Sciences and Wellness, University of Arizona, Tucson, AZ, United States; ^4^Knight Cancer Institute, School of Medicine, Oregon Health & Science University, Portland, OR, United States

**Keywords:** Hispanic Americans, prostatic neoplasms, exercise, informal caregivers, community-based participatory research, implementation science

## Abstract

**Background:**

Cancer disparities exist for Hispanic men with prostate cancer and their caregivers that could be reduced through exercise. Exercising Together^©^ is a six-month, evidence-based dyadic resistance training program that promotes teamwork between prostate cancer survivors and their spouses to improve physical, mental, and relational health outcomes. The purpose of this study was to elicit feedback and recommendations from stakeholders on the Exercising Together^©^ intervention to inform the cultural adaptation of this program for Hispanic men with prostate cancer.

**Methods:**

We conducted a virtual Community Engagement Studio (V-CES) with community expert stakeholders representing the Hispanic and cancer care communities in Southern Arizona. The V-CES process included orientation, presentation of the research, guided discussion, and evaluation. The V-CES was audio recorded, transcribed, and rapidly analyzed to identify actionable feedback and contextual adaptations.

**Results:**

Nine stakeholders (6/9 male; 5/9 Hispanic) completed all V-CES activities. Through stakeholder engagement and feedback from the V-CES, adaptations to the original Exercising Together^©^ intervention included: (1) inclusion of the cancer survivor’s identified caregiver, who may not be a spouse; (2) availability in English and Spanish; (3) shortening the intervention to 3 months; (4) remote delivery of the intervention; and (5) incorporation of low burden procedures.

**Conclusion:**

Findings from our V-CES informed the adaptation of a culturally relevant dyadic progressive resistance training program for Hispanic men with prostate cancer and their caregivers.

## 1 Introduction

Prostate cancer is the most common cancer among Hispanic men in the United States (US) (Miller et al., 2021; Siegel et al., 2023). Compared to non-Hispanic white men, Hispanic men have a higher prevalence of prostate cancer and lower prostate cancer-specific mortality. However, they also face a higher risk of comorbidities and a lower quality of life (Penedo et al., 2006; Dominguez et al., 2015). Hispanic men are disproportionately diagnosed with prostate cancer at advanced stages (Hoffman et al., 2001), which often requires more aggressive treatment with more significant side effects (Stern, 2020). Prostate cancer treatment can lead to decreased muscle mass, metabolic dysfunction, and other adverse health effects that contribute to the burden of morbidity and mortality (Galvão et al., 2008; Winters-Stone et al., 2017; Gebauer et al., 2019; Lopez et al., 2021).

Cancer caregivers also experience subsequent poor health outcomes and unmet needs (Siefert et al., 2008; Badger et al., 2019; Skiba et al., 2022). For married cancer survivors, the spouse often takes on the role of informal caregiver, providing approximately half of the unpaid care to the survivor (Kent et al., 2016; Skiba et al., 2021). Hispanic cultural values like *machismo* (perceived masculine qualities), *familismo* (a strong obligation to do what is best for the family rather than the individual), and *marianismo* (perceived feminine qualities) have been connected to cancer caregiving (Badger et al., 2019). These cultural values influence the choice of a caregiver, who may be a spouse or a different family member such as a sibling, adult child, or parent (Kent et al., 2016; Badger et al., 2019).

Current guidelines recommend that all cancer survivors engage in regular exercise (Campbell et al., 2019; Ligibel et al., 2022; Rock et al., 2022). Exercise improves treatment tolerance, metabolic biomarkers, and physical function while preserving lean muscle mass, reducing impairment, and decreasing the risk of disability (Winters-Stone et al., 2015; Ligibel et al., 2022). However, adherence to these guidelines remains low among Hispanic adults and cancer survivors (Arredondo et al., 2016; Skiba et al., 2021). Importantly, recent evidence suggests that the inclusion of a cancer caregiver in interventions may better improve adherence to current guidelines and cancer outcomes (Badr et al., 2019). Presently, few dyadic interventions exist for Hispanic cancer survivors and none aim to improve exercise behaviors tailored to Hispanic men with prostate cancer (Rush et al., 2015; Cho et al., 2020; Badger et al., 2021; Crane et al., 2021; Winters-Stone et al., 2021a,b).

Exercising Together^©^ is an evidence-based dyadic progressive functional resistance training program designed to promote teamwork between cancer survivors and their spouses (Winters-Stone et al., 2012, 2021a,b). The core exercise intervention focuses on muscle groups and movements pivotal for activities of daily living and emphasizes teamwork by incorporating tandem versions of exercises that require collaboration and communication (Winters-Stone et al., 2021a). Dyadic group exercise classes supervised by certified exercise instructors meet twice a week for six months. Notably, Exercising Together^©^ has been shown to be highly feasible and effective in improving physical, mental, and relational health in mostly non-Hispanic white prostate cancer survivors and their spouses (Winters-Stone et al., 2016, 2021b). Despite these promising results, an important unmet need remains to better understand the cultural context and population preferences to better engage Hispanic men with prostate cancer in exercise interventions for the greatest impact on health and cancer outcomes. This paper describes the key findings gathered from a virtual Community Engagement Studio to inform the cultural adaptation of Exercising Together^©^ for Hispanic men with prostate cancer.

## 2 Methods

### 2.1 Community engagement studio

The Community Engagement Studio (CES) model was developed by the Meharry-Vanderbilt Community Engaged Research Core (CERC). CES provides a structured approach for collecting feedback to inform research design, implementation, and dissemination of the existing interventions (Joosten et al., 2015, 2018). Briefly, the CES model includes orientation to the process, presentation of the research, guided discussion, and evaluation. For the present study, we followed the procedures detailed in the Virtual Community Engagement Studio Toolkit (Maternal Mental Health Research Collaborative, 2021).

The virtual CES (V-CES) was conducted with expert stakeholders representing the Southern Arizona Hispanic and cancer care communities on 23 May 2023. Community expert stakeholders were recruited through existing networks and community outreach events, and were provided access to information about the Exercising Together^©^ intervention through a private Google Drive folder prior to the V-CES.

A bicultural and bilingual facilitator led the V-CES which was conducted in English due to the limitations of existing intervention materials, which were only available in English at the time of the V-CES. The beginning of the V-CES included a presentation of the research and goals by the site principal investigator. In the context of Hispanic men with prostate cancer, five questions were asked during the V-CES: (1) What are your thoughts about a group exercise program such as Exercising Together^©^? (2) How can Exercising Together^©^ be more inclusive and relevant to population needs? (3) What would you change about Exercising Together^©^? (4) Why would you choose to participate in or refer a patient to Exercising Together^©^? (5) What health outcomes are most important to you?.

The V-CES was conducted virtually using Zoom for Healthcare software (Zoom Video Communications, Inc. San Jose, CA), lasted 90 min, and was audio recorded. Notes were taken by the facilitator during the V-CES to ensure clarity of interpretation. After the V-CES, an evaluation survey was sent to the stakeholders asking for additional thoughts about the Exercising Together^©^ program including 10 items related to cultural competence on the curriculum (Lie et al., 2008) in addition to their experiences and contributions from participating in the V-CES. Stakeholders were compensated USD 25 for their time at the completion of the V-CES activities.

### 2.2 Analysis

Audio recordings were transcribed verbatim as a service of the Behavioral Measurement and Instrument Shared Resource at the University of Arizona Cancer Center. Transcripts for all questions were thematically analyzed using framework guided rapid analysis procedures that have been shown to produce valid results and identify actionable suggestions (Gale et al., 2019). A codebook was developed based on the Typology of Adaptation Approaches and the Pathway for Adaptation as proposed by Davidson and colleagues (Davidson et al., 2013) to guide the analysis and identify actionable feedback. Exemplary quotes and key points were highlighted. Two coders independently reviewed the transcripts and summarized stakeholder feedback using a standardized template built in Microsoft Excel Version 16.75.2 (Microsoft Corporation, Redmond, WA). Two coders had 66.7% agreement (κ = 0.64, *p* < 0.001) prior to discussion to reach consensus on a final codebook. Actionable feedback was mapped following the RESET (Relevance, Evidence Base, Stage of Intervention, Ethnicity, Trends) (Davidson et al., 2013) decision-making tool for identifying contextual adaptations to Exercising Together^©^.

## 3 Results

### 3.1 Community expert stakeholders

A total of 12 stakeholders were recruited to participate. Three became unavailable at the scheduled time of the V-CES, leaving nine to complete all V-CES activities. Among these nine stakeholders, the mean age was 47.9 ± 16.8 years, six were men, three were women, and five identified as Hispanic. Data on Hispanic subgroups (e.g., Mexican origin) were not collected. Stakeholder representation included two clinical oncology providers, four community health workers, one preclinical cancer researcher, one family member of a prostate cancer survivor, and one prostate cancer survivor.

### 3.2 Stakeholder feedback on the exercising together^©^ intervention

The dyadic group exercise component of the intervention was viewed primarily as a strength, facilitating social support, and resonating with cultural values anchored in *familismo* and *marianismo*. A minor concern was whether a spousal caregiver would be available to participate with a survivor. However, the commitment of the spouse or family was seen as a crucial motivator for participation. Stakeholders highlighted that those who have undergone androgen deprivation therapy (ADT) and are frail may derive the greatest benefit. Additionally, underlined by *machismo*, it was emphasized that the intervention should be sensitive to the private and sometimes stigma surrounding prostate cancer within the Hispanic culture and that a focus on the community, strength-building, and self-care components would be essential.

The major strengths were the guided and supervised exercise classes by certified trainers in a group setting while concerns were related to cultural relevance, specifically, Spanish language availability and ethnically and gender matched interventionists. A shorter intervention period was perceived to have greater acceptability. In-person participation, especially at a cancer clinic, was considered to have potentially high costs and to be time-consuming for participants, particularly those living in more rural areas. Therefore, the ability to participate remotely from home was a preferred option. While one stakeholder expressed concerns over health literacy and Internet access for patients, remote delivery of the intervention appeared to be feasible and acceptable to stakeholders overall and appeared to have the potential to promote participant engagement and retention. Recruitment of participants would be best achieved by partnering with oncology providers to refer patients to the intervention, partnering with patient advocates and support groups, and facilitating buy-in from caregivers, especially spousal caregivers. Sample quotes mapped to RESET (Davidson et al., 2013) are below.

#### 3.2.1 Relevance: is this health promotion topic relevant to the target population?

- “Exercise is something that especially in prostate cancer is really great… exercise has been a missing component [in the clinic].”
- “Exercise helps with, you know, hot flashes, help with building back muscle. They all feel they are going to lose muscle. So, building muscle, you know, fatigue improvement.” [*SIC*]
- “Plenty of patients that need this kind of intervention early on.” [*SIC*]
- “So maybe even writing kind of like an informal prescription to refer people to this program saying, you know, part of your prescription is that you join an exercise program because it’s part of your treatment and then you make it a holistic thing.” [*SIC*]
- “It’s going to be important for the person leading the group to make that group feel comfortable so that they can return and continue.” [*SIC*]
- “Personal benefits can come from [Exercising Together^©^], benefits that actually go beyond just cancer care, especially with the communication piece.” [*SIC*]
- “If a person is putting in all this effort, they would like to see at least in some kind of way how they have progressed over time to see how they have benefited from it, how much stronger they have gotten, not only physically but mentally.” [*SIC*]

#### 3.2.2 Evidence base: what is the best intervention to address this health issue in this population?

- “Finding the right exercise, something that people enjoy, and it does not feel like it’s something that they have to do, but something that they enjoy doing.” [*SIC*]
- “[Participants will be] successful just because they start exercising together. So it becomes more of a peer support type of thing with the exercise included.” [*SIC*]
- “Visualizes what the exercises would be… if the participants would like to revisit these exercises on their free time to kind of practice getting a better form, exercising properly, I think that would be extremely helpful for this.” [*SIC*]

#### 3.2.3 Stage of intervention: what stage(s) of the intervention should be adapted?

- “Just kind of being mindful about those type of barriers [social determinants of health] and just trying to meet people where they are at because I think we do not want to burden them anymore. You know, they are already dealing with all this treatment and navigating the system… and it’s complicated enough already.” [*SIC*]
- “Aside from that, would also be just any kind of language changes…If a person is not English-speaking or they have very low literacy, I think probably just adjusting to that would be beneficial.” [*SIC*]
- “Sometimes [we] associate the cancer center with cancer and might not want to use the center as a place for this kind of activity simply because there’s some bad associations.” [*SIC*]
- “The kind of relationship with the clinic and you know, things that may happen there that they do not want to be associated.” [*SIC*]
- “I think a staying away from the clinical setting would be nicer just for a peace of mind and from trauma that maybe causes coming back to a cancer center and having that, memories of getting chemo there or your treatment.” [*SIC*]

#### 3.2.4 Ethnicity: what elements of ethnicity are most important to consider for this population?

- “A lot of times, you know, the community aspect of it and the encouragement really helps people if they are having a challenge.” [*SIC*]
- “So, you know, in Spanish ‘*El Doctor me dijo tengo hacer esto*’ is like ‘*Tengo que*. *I have t*o.’”
- “Being inclusive, reaching out to the community. This has to be translated [to Spanish]. It has to be in lay terms.” [*SIC*]
- “I’ve heard many times that the guys would not even be in the group except their wives said, ‘*You get yourself into that group*.’” [*SIC*]
- “It’s a plus to add to the exercise and get that peer support as well. Now, if you include a family member, that would be even better. And if it’s the wife like we have mentioned before, maybe you would have a better chance of getting patients in.” [*SIC*]
- “Hispanic men do not tend to tell you that they have problems. They have, you know, the wife is the one that will expose them in front of [the doctor]. So you really will have to invite the wife or the daughter or the son.” [*SIC*]

#### 3.2.5 Trends: What are the shifting trends in this population?

- “Any type of community gathering place where you would have individuals who have the means and capabilities to gather together.” [*SIC*]
- “Going online… assuming one, they are proficient at their age, two, they have Internet, three, they have capacity and four, they will follow along.” [*SIC*]
- “If it was by [videoconferencing], then [we] would be able to see [exercise instructor] doing the exercises and could make the corrections at the time.” [*SIC*]
- “The exercises you can do at home.” [*SIC*]
- “Make it really practical.” [*SIC*]

### 3.3 Cultural competence

From the V-CES evaluation surveys, 86% of stakeholders agreed that the Exercising Together^©^ program identifies community beliefs and health practices, respects patients’ cultural beliefs, and effectively factors in patients’ cultural, social, and medical histories. Additionally, 71% of stakeholders agreed that the intervention elicits information in a family-centered context. When asked about how they felt about their contributions to the research project, 38% strongly agreed that they provided feedback on the feasibility and appropriateness of the project and ideas for recruiting research participants. Half of the stakeholders agreed that they increased the researcher’s understanding of the population.

### 3.4 Integrating stakeholder feedback into culturally relevant adaptations

Adaptations made to Exercising Together^©^ to provide a culturally relevant intervention include expanding from a sole focus on spousal caregivers to involve a survivor-designated caregiver from any relationship type. Intervention materials and delivery will be available in English and Spanish. The duration of the intervention will be reduced from 6 months to 3 months and the intervention will be delivered remotely. Remote delivery will include supervised online group exercises led by bilingual English/Spanish interventionists who are ethnically and gender matched. We will also incorporate low burden assessment procedures for identified important health and aging-related outcomes such as cancer-related symptoms and physical function. These adaptations are expected to further reduce travel time and burden for individuals residing in rural communities and increase the reach of the intervention. Fidelity to the original intervention is retained through the dyadic approach to resistance training, which builds teamwork and communication and by having all study exercise classes led and supervised by a certified exercise instructor trained in the intervention protocol to ensure proper progression, tolerability, and safety. The adapted Exercising Together^©^ program will be pilot tested for feasibility and acceptability in a three-month single-arm trial among a sample of Hispanic men with prostate cancer and their caregivers.

## 4 Discussion

The purpose of this study was to conduct a V-CES to inform cultural adaptations of an existing evidence-based dyadic intervention prior to pilot testing in a feasibility trial. We utilized a V-CES to engage community expert stakeholders in Southern Arizona to better understand population needs and preferences and guide the cultural adaptation of Exercising Together^©^, a dyadic resistance training intervention, for Hispanic men with prostate cancer and their caregivers. Actionable stakeholder recommendations for increasing the reach, acceptability, and cultural relevance of the intervention identified from the V-CES were the inclusion of diverse family caregivers, ensuring the availability of all materials and intervention activities in Spanish, and incorporating low burden study procedures.

The CES model has been used in other studies to inform modifications needed to make interventions more relevant, feasible, acceptable, and sustainable in target communities. Recently, the CES has been successfully used to gain insights on participation in cancer screening research among the Hispanic populations in rural communities (Currier et al., 2023) and to adopt a social incentive intervention to increase physical activity among urban minorities and older adults (Scheffey et al., 2022). Our V-CES aligned with established cultural adaptation processes for health interventions (Bernal et al., 1995; Barrera et al., 2013).

This study contributes to the limited literature on exercise program preferences among Hispanic men with cancer. To date, there has only been one focus group study of 14 men with advanced prostate cancer (of whom 21% identified as Hispanic) to identify preferences for digital health interventions (Wang et al., 2023). Key themes identified from this study included social support and family identity in group exercise classes with social support being a motivating factor for participation. Separate research focusing on overweight Hispanic men also recognized social support and family involvement including spousal recruitment as intervention strategies for weight management (Garcia et al., 2017). Among low-income Hispanic men exercise was seen as a way to maintain physical health with a preference for interventions delivered by trained professionals and family involvement in the program (Portacio et al., 2018). These findings are similar to those from our V-CES.

### 4.1 Strengths and limitations

A key strength of this study was the use of a CES model that effectively increased community engagement in clinical research and elicited feedback from community expert stakeholders on the cultural adaptation of an existing evidence-based intervention. Compared to traditional qualitative research methods such as interviews or focus groups, CES provides researchers with a platform to engage community members in the research process and gather feedback to quickly adapt interventions for the target community and context. As such, our V-CES was designed in a way to gather feedback on the Exercising Together^©^ program rather than gain a comprehensive understanding of lived experiences. We applied qualitative data analysis procedures mapped to an existing framework to guide the adaptation of the intervention based on stakeholder feedback and recommendations.

A limitation of the CES model is the potential for sampling bias in the selection of expert community stakeholders. While our stakeholder cohort was representative of the Hispanic and cancer care communities in Southern Arizona, it may not be entirely representative of all Hispanic male cancer survivors and caregivers in the region. Consequently, our findings may not represent the perspectives of monolingual Spanish speaking participants and may not be generalizable to other subcultures or populations.

### 4.2 Conclusion

Utilizing a V-CES, we collected stakeholder feedback on how to culturally adapt Exercising Together^©^ for Hispanic cancer survivors to ensure alignment with cultural values, representation of the population, and consideration of communication and intervention delivery preferences. Researchers adapting other evidence-based interventions to be culturally relevant may find the use of a CES model beneficial.

## Data availability statement

The raw data supporting the conclusions of this article will be made available by the corresponding author upon reasonable written request without undue reservation.

## Ethics statement

All procedures performed in studies involving human participants were in accordance with the ethical standards of the institutional and/or national research committee and with the 1964 Helsinki declaration and its later amendments or comparable ethical standards. Although Community Engagement Studios are not explicitly human subjects research and often may not require ethical approval, as stakeholders are considered consultants rather than research participants, this study was approved by the Institutional Review Board at the University of Arizona (STUDY00002341). All stakeholders provided their written informed consent to participate in this study.

## Author contributions

MS: Conceptualization, Funding acquisition, Methodology, Supervision, Writing – original draft. TB: Conceptualization, Writing – review & editing. DG: Conceptualization, Writing – review & editing. FC: Writing – review & editing. KW-S: Conceptualization, Methodology, Resources, Writing – review & editing.
